# Supplementary material for: Development and validation of psychosocial determinants measures of physical activity among Iranian adolescent girls
Source: BMC Public Health. 2008 May 7;8:150. doi: 10.1186/1471-2458-8-150 (PMC2397400; doi:10.1186/1471-2458-8-150)
Supplement: Additional file 1 — Final translation of psychosocial determinants of physical activity questionnaires. Translation and back translation procedure was used to develop culturally equivalent questionnaires. Self – efficacy of physical activity. There are many barriers in the way of physical activity. Mark the extent of your capability for doing physical activity in the following situations. Answer all the questions. 1. Can you do exercise or physical activity even when you are sad or under stress? 2. Can you dedicate a specific time for doing exercise or physical activity on most days of the week?. 3. Can you do exercise or physical activity even when your family or friends ask you to do something else?. 4. Can you get up early to do your exercise or physical activity even on the weekends?. 5. Can you do your exercise or physical activity even when you have a lot of homework to do?. 6. Can you do your exercise or physical activity even when the weather is hot or it is rainy?. Family support of physical activity. 1. How many days a week do your family members watch your exercise or physical activity?. 2. How many days a week do your family members encourage you to do exercise or physical activity?. 3. How many days a week do your family members provide you transportation go to a place for doing exercise or physical activity?. 4. How many days a week do your family members do their exercise or physical activity with you?. Friend support of physical activity. 1. How many days a week do your friends encourage you to do exercise or physical activity?. 2. How many days a week do your friends do their exercise or physical activity with you?. 3. How many days a week do your friends tease you for doing well your exercise or physical activity?. 4. How many days a week do your friends ask you to walk or bike from your house to the school or their houses?. 5. How many days a week do your friends tell you that you are doing well your exercise or physical activity?. 6. How many of your 5 closest frien [file 1471-2458-8-150-S1.doc]

Additional file
File format: DOC
Title: Final translation of psychosocial determinants of physical activity questionnaires

Description: Translation and back translation procedure was used to develop culturally equivalent questionnaires

**Self – efficacy of physical activity**

There are many barriers in the way of physical activity. Mark the extent of your capability for doing physical activity in the following situations. Answer all the questions.

1. Can you do exercise or physical activity even when you are sad or under stress?
2. Can you dedicate a specific time for doing exercise or physical activity on most days of the week?
3. Can you do exercise or physical activity even when your family or friends ask you to do something else?
4. Can you get up early to do your exercise or physical activity even on the weekends?
5. Can you do your exercise or physical activity even when you have a lot of homework to do?
6. Can you do your exercise or physical activity even when the weather is hot or it is rainy?

**Family support of physical activity**

1. How many days a week do your family members watch your exercise or physical activity?
2. How many days a week do your family members encourage you to do exercise or physical activity?
3. How many days a week do your family members provide you transportation go to a place for doing exercise or physical activity?
4. How many days a week do your family members do their exercise or physical activity with you?

**Friend support of physical activity**

1. How many days a week do your friends encourage you to do exercise or physical activity?
2. How many days a week do your friends do their exercise or physical activity with you?
3. How many days a week do your friends tease you for doing well your exercise or physical activity?
4. How many days a week do your friends ask you to walk or bike from your house to the school or their houses?
5. How many days a week do your friends tell you that you are doing well your exercise or physical activity?
6. How many of your 5 closest friends do their physical activity regularly?

**Positive and negative of physical activity**

The following sentences are different beliefs about physical activity.

Please choose the degree which indicates the importance of each sentence to you whenever deciding whether or not to do your physical activity.

1. I would feel embarrassed if people saw me doing physical activity.
2. Physical activity helps me to keep fit.
3. My parents would become happy if I do physical activity.
4. I have to learn a lot of things to be able to do physical activity.
5. If I do physical activity I would feel better about myself.
6. I would need too much help from my parents to be able to do physical activity.
7. Physical activity and exercise makes me unpleasant feeling.
8. I would have fun doing physical activity or playing sports with my friends.
9. If I do physical activity I would have more energy.
10. Physical activity reduces the time I spend for being with my friends.

**The strategies to change physical activity**

The followings are activities/ thoughts, and feelings people use to help them change their physical activity. Think about yourself similar cases that you experience or have experienced during the past month then indicate HOW OFTEN you do each of the followings:

1. I look for information about physical activity or sports.

2. I keep the account of my physical activity.

3. I find out the ways to overcome the obstacles of doing physical activity.

4. I think about the effect of facilities (such as having sports equipments at home or in the near by park) on the amount of my physical activity.

5. To be more physically active I keep things at home to remind me that.

6. I encourage myself for doing physical activity or exercise.

7. I do things to make physical activity more enjoyable.

8. I think about the benefits I get from the physical activity or exercise.

9. I think about the benefits of physical activity than its troubles.

10. I say to myself about the positive sides of physical activity.

11. When my physical activity plans are stopped for a while,

I tell myself I can start again and become active.

12. I have a friend or family member who encourages me to do physical activity.

13. I try different kinds of physical activity to have more options to choose from.

14. I set goals for doing physical activity.

15. I consider alternative plans to assure myself of having physical activity.

**Environmental factors of physical activity:**

Define your attitude about your life environment and indicate how much do you agree with the following sentences?

1. There is enough sports equipment at home to use for physical activity.
2. Walking or jogging is difficult around my house, because of traffic, lack of side walks, dogs, gangs, and so on.
3. It is possible to access playgrounds, parks or gyms near to my home for doing physical activity or exercise.
4. It is safe to walk or jog during the day around my home.

**Physical activity enjoyment**

1. I enjoy doing physical activity or exercise.

**Recreation choices of physical activity**

2. Which of the following activities do you usually choose to spend on your leisure time?

(1) I almost always choose the activities like watching T.V, studying, listening to music, or working with computer.

(2)I usually most always choose the activities like watching T.V, studying, listening to music, or working with computer.

(3) It is possible to choose active entertainments as much as inactive ones.

(4) I usually choose the activities like riding, bike, skating, and games played, outside or active sports.

(5) I almost always choose the activities like riding, bike, skating, and games played, outside or active sports.
